# Unexpected results in Chernozem soil respiration while measuring the effect of a bio-fertilizer on soil microbial activity

**DOI:** 10.12688/f1000research.12936.2

**Published:** 2017-12-18

**Authors:** Gabriela Bautista, Bence Mátyás, Isabel Carpio, Richard Vilches, Karina Pazmino

**Affiliations:** 1 Department of Agricultural Chemistry and Soil Sciences, University of Debrecen, Debrecen, Hungary; 2 Grupo de Investigación Mentoria y Gestión del Cambio, Universidad Politécnica Salesiana, Cuenca, Ecuador; 3 Grupo de Investigación en Ciencias Ambientales, Universidad Politécnica Salesiana, Quito, Ecuador; 4 Ingeneria Ambiental, Universidad Politécnica Salesiana, Quito, Ecuador; 5 Grupo de Innovación Educativa UPS en Ciencas Básicas, Universidad Politécnica Salesiana, Quito, Ecuador

**Keywords:** bio-fertilizer, soil respiration, Chernozem, OxiTop

## Abstract

The number of studies investigating the effect of bio-fertilizers is increasing because of their importance in sustainable agriculture and environmental quality. In our experiments, we measured the effect of different fertilizers on soil respiration. In the present study, we were looking for the cause of unexpected changes in CO2 values while examining Chernozem soil samples. We concluded that CO2 oxidizing microbes or methanotrophs may be present in the soil that periodically consume CO2 . This is unusual for a sample taken from the upper layer of well-ventilated Chernozem soil with optimal moisture content.

## Introduction

The soil can be characterized by physical, chemical and microbiological properties ^1– 4^. The quantitative (microbial biomass, number of bacteria) ^5, 6^ and qualitative (enzymatic activity, soil respiration) ^7, 8^ microbiological properties of the soil greatly contribute to the impact analysis of land use ^9– 11^, nutrition ^12^ and soil management ^13^. Research related to the benefits of microbes as biofertilizer has become increasingly important in the agricultural sector. This is due to the possibility of achieving higher crop yields while minimizing negative impact on the environment. It is well known that bio-fertilizers increase plant yield and improve soil fertility ^14– 16^. Soil respiration is an important indicator of soil microbial activity ^17, 18^. In our experiments, we measured the effect of different chemicals ^19– 22^ and a bio-fertilizer on soil microbial activity, using both well-established and novel methods under laboratory conditions. We present some unexpected results from a setup in which Chernozem soil samples were examined.

## Methods

### Sampling site

A total of 24 soil samples were collected near Debrecen, Hungary, on the 19th April 2016, from an upper layer (0–20 cm) of Chernozem soil (47°33’ 55.36” N; 21°28’ 12.27” E).

### Treatment

The phylazonit bio-fertilizer (produced by Phylazonit.Ltd, Hungary) with the following composition: *Bacillus megaterium*, *Bacillus circulans*, *Pseudomonas putida*, was tested (15 l/ha) in an optimized ratio for soil injection. Number of bacteria: 10 ^9^ *piece/cm* ^3^.

### Soil properties

Soil moisture content was determined gravimetrically, drying the soil at 105°C for 24 hours according to Klimes-Szmik’s method (1970) ^23^. Silt and clay fractions were measured by the settling method ^24^. We measured the Arany-type plasticity index according to Stefanovits (1975) ^25– 27^, while the minimal water capacity and soil texture were determined by Klimes-Szmik’s method ^23^. To measure the chemical properties of the soil, the samples were sieved through 2mm mesh and pre-incubated at 25°C for 72 hours. Soil pH in distilled water and in 1M potassium chloride KCl (soil/water, 1/2.5, w/w) were determined according to Buzás (1988) ^24^. The electrical conductivity (EC) (soil/water, 1/5, w/w) was then determined with a glass electrode according to Kong *et al*, 2013 ^28^. The hydrolytic acidity (y1) was measured according to Buzás (1988) ^24^, while the concentration of NO _3_^−^ -N was determined according to Felföldy (1987) ^29^. Total nitrogen was determined according to Kong *et al*. (2013) ^28^. Nitrate exploration was carried out after 14 days incubation according to Felföldy (1987) ^29^. We determined AL-P _2_O _5_ and ALK _2_O based on Szegi’s method (1979) ^30^. The humus content was determined using potassium dichromate according to Székely (1988) ^31^. Total number of bacteria was counted in bouillon agar using the plate dilution method (Szegi, 1979) ^30^. We measured the organic carbon concentration in K _2_SO _4_ extract, following the protocol in Székely *et al*. (1988) ^31^. Microbial biomass carbon (MBC) was measured using the chloroform fumigation-extraction method. Soil samples were fumigated by adding alcohol-free chloroform at 25°C for 24 hours. The fumigated and unfumigated soil samples were extracted with 50 ml 0.5 M potassium sulfate (K _2_SO _4_) according to Vance *et al*. (1987) ^32^. The following formula was applied to calculate the MBC (Kong *et al*., 2013) ^28^: 

                                                                                            MBC = 2.22 x EC

where EC = organic C extracted from fumigated soils – organic C extracted from unfumigated soils ( Table 1).

**Table 1.** Average values for a number of different soil properties.

Soil property	Value	Unit	Protocol
Silt and clay fraction	37.48	%	BuzásI. (1988): Manual of Soil and Agrochemical Analysis Vol.1. (in Hungarian). INDA 4231 Kiadó. Budapest.
Hygroscopicity	2.23	hy	BuzásI. (1988): Manual of Soil and Agrochemical Analysis Vol.1. (in Hungarian). INDA 4231 Kiadó. Budapest.
Arany-type of plasticity limit	39	KA	Szegi
Moisture content	19–21	%	Szegi
Hydrolytic acidity	5.94	y1	BuzásI. (1988): Manual of Soil and Agrochemical Analysis Vol.1. (in Hungarian). INDA 4231 Kiadó. Budapest.
organic-C	1.4	%	Székely
Nitrate-N	7.4	mg/kg	Hayashi A, Sakamoto K, Yoshida T 1997: A rapid method for determination of nitrate in soil by hydrazine reduction produce. Jpn. J. Soil Sci. Plant Nutr.,68, 322
Total-N	2.6	mg g–1 D.S	
AL-soluble P	48.6	P2O5 mg/kg	Szegi
AL-soluble K	222	K2O mg/kg	Szegi
pH (H2O)	6.8	pH	BuzásI. (1988): Manual of Soil and Agrochemical Analysis Vol.1. (in Hungarian). INDA 4231 Kiadó. Budapest.
pH (KCl)	6.1	pH	BuzásI. (1988): Manual of Soil and Agrochemical Analysis Vol.1. (in Hungarian). INDA 4231 Kiadó. Budapest.
Topsoil	80–90	cm	Szegi
Soil texture	Loam		BuzásI. (1988): Manual of Soil and Agrochemical Analysis Vol.1. (in Hungarian). INDA 4231 Kiadó. Budapest.
Minimal water capacity	26.22	VKmin	Szegi
Humus content	2.81	%	BuzásI. (1988): Manual of Soil and Agrochemical Analysis Vol.1. (in Hungarian). INDA 4231 Kiadó. Budapest.
Total number of bacteria	9.59	1.000.000 colony/g	Szegi
Nitrate exploration	34.28	mg/kg	Felföldy
Microbial biomass carbon	333	mg/kg	Vance ED, Brookes PC, Jenkinson DS 1987: An extraction method for measuring soil microbial biomass-C. Soil Biol. Biochem.,19, 703–707.

### Soil respiration

**Figure 1.**
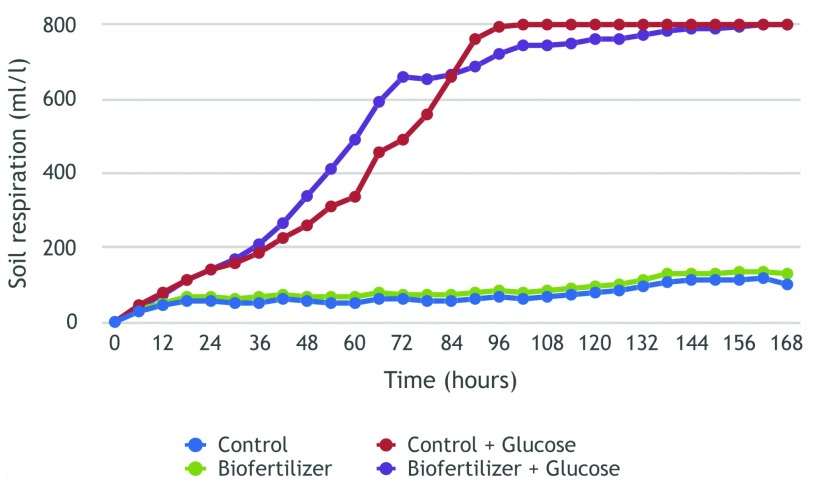
Differences in *CO*_2_ production of treated and control soil samples. The induced method was carried out so that the difference between the results of control and the treated soil samples could become detectable sooner. Glucose was applied as inducer. As expected the *CO*_2_ values increase or stagnate.

The experimental design was completely randomized, treatments were incubations (25°C). An OxiTop OC110 respirometer was used to quantify the release and capture of CO _2_ that is automatically determined by the device after the biological oxygen demand (BOD) required for the degradation of organic matter has been measured. We used a 500 ml glass bottle system following the instruction manual ( https://www.wtw.com/en/service/downloads/operating-manuals.html). 10g of soil sample were placed into OxiTop flasks, and capped with the sensor heads according to Barrales-Brito *et al*. (2014) ^33^. 2.5g of CO _2_ absorber (sodalime) were then added to a tank to absorb the generated CO _2_^33^. An induced method was also used, in which 0.1g glucose was added to the soil samples. Each treatment was replicated four times. As Figure 1 shows, four samples were always measured in parallel: Absolute control (does not contain fertilizer, nor added glucose), Induced control (contains added glucose), Treated (contains bio-fertilizer) and Induced treated (contains bio-fertilizer and glucose). The Oxitop automatically provides the values related to CO _2_ production according to the pressure change measured by its sensor (there is no need to carry out titrations or any additional work).

## Results

**Figure 2.**
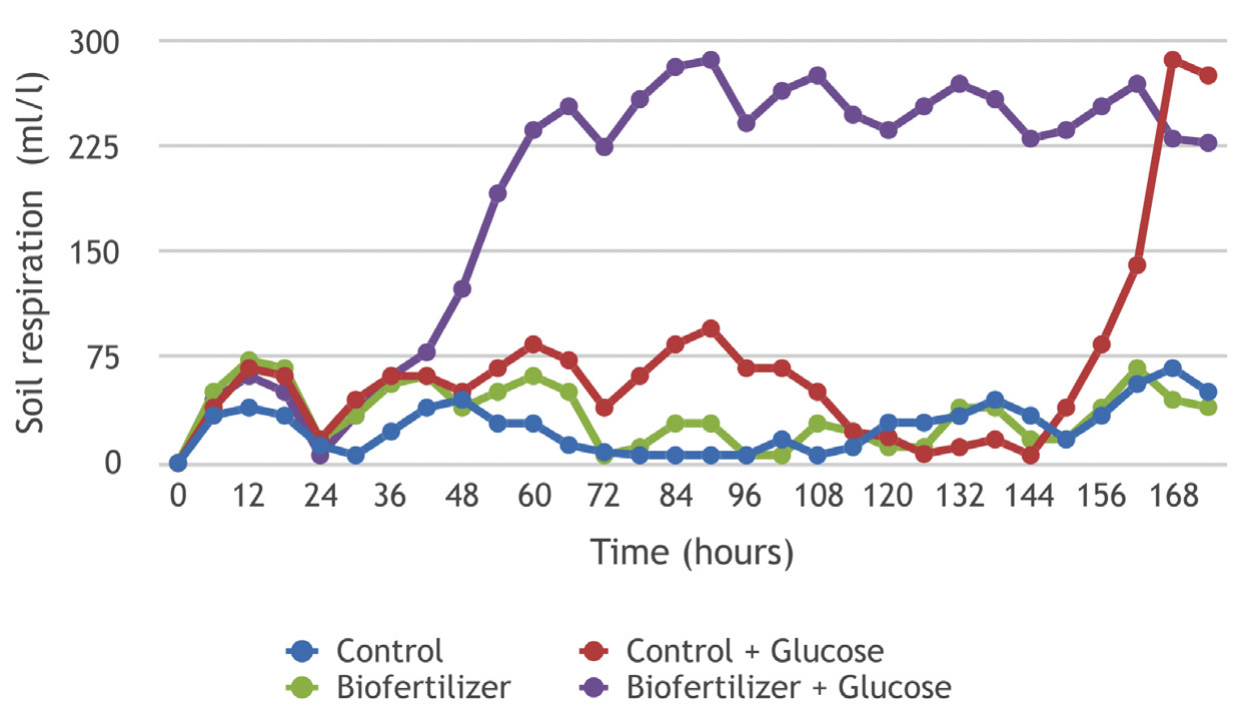
This sample shows *CO*_2_ values periodically decreasing in all conditions. After examining the Oxitop device’s operation, this pattern became more interesting to us, as the device quantifies *CO*_2_ production by measuring BOD required for the degradation of organic matter. From the decreasing *CO*_2_ values, we conclude that there was oxygen production and/or *CO*_2_ consumption in the Oxitop bottles.

The treated samples produced more *CO*_2_ than the controls, as expected ( Dataset 1). Each repeat with the exception of one showed increasing *CO*_2_ values ( Figure 1), as the pressure continuously decreased in the bottle due to gas (oxygen) consumption. One sample produced unexpected results ( Figure 2). In the first 12 hours, the treated samples produced more *CO*_2_ than the controls in each measurement. Following this, a fluctuation in the values was observed.

Average values of produced CO2 (ml/l) with different treatments. ’Control’ does not contain fertilizer, nor added glucose. ’Control+Glucose’ contains 0,1 g of added glucose. ’Biofertilizer’ contains Phylazonit biofertilizer. ’Biofertilizer+Glucose’ contains Phylazonit biofertilizer and 0,1 g of added glucose Click here for additional data file. Copyright: © 2017 Bautista G et al. 2017 Data associated with the article are available under the terms of the Creative Commons Zero "No rights reserved" data waiver (CC0 1.0 Public domain dedication).

Comparison of produced CO2 (ml/l) in the sample in which unexpected (periodically decreasing CO2) values can be observed. ’Control’ does not contain fertilizer, nor added glucose. ’Control+Glucose’ contains 0,1 g of added glucose. ’Biofertilizer’ contains Phylazonit bio-fertilizer. ’Biofertilizer+Glucose’ contains Phylazonit bio-fertilizer and 0,1 g of added glucose Click here for additional data file. Copyright: © 2017 Bautista G et al. 2017 Data associated with the article are available under the terms of the Creative Commons Zero "No rights reserved" data waiver (CC0 1.0 Public domain dedication).

## Discussion

In a closed system where the pressure decreases due to oxygen consumption, the values of CO2 production must increase or stagnate with the passage of time, but this was not the case with one of the samples ( Figure 2). Here, a decrease in *CO*_2_ occurred ( Dataset 2). The following possible explanations were excluded:

- Presence of algae: there was no light in the incubator, so there was no photosynthesis.
- Changing pressure caused by changing temperature: the temperature was constant in the setup.
- Absorption by the water in the sample: all other samples that produced increasing amount of *CO*_2_ had the same or comparable moisture content.

One reason that seemed more likely was that *CO*_2_ oxidizing microbes or methanotrophs may have been present in the soil, using the produced *CO*_2 _periodically. This is unusual, since most of the studies report the presence of these bacteria in seawater ^34^, paddy fields ^35^ or industrial processes ^36^ and not in well-ventilated Chernozem soil. Further genomics research could detect the bacterial strains that consumed the *CO*_2_ in this soil.

## Data availability 

Dataset 1: Average values of produced *CO*_2_ (ml/l) with different treatments. ’Control’ does not contain fertilizer, nor added glucose. ’Control+Glucose’ contains 0,1 g of added glucose. ’Biofertilizer’ contains Phylazonit bio-fertilizer. ’Biofertilizer+Glucose’ contains Phylazonit bio-fertilizer and 0,1 g of added glucose. DOI, 10.5256/f1000research.12936.d182663 ^37^.

Dataset 2: Comparison of produced *CO*_2_ (ml/l) in the sample in which unexpected (periodically decreasing *CO*_2_) values can be observed. ’Control’ does not contain fertilizer, nor added glucose. ’Control+Glucose’ contains 0,1 g of added glucose. ’Biofertilizer’ contains Phylazonit bio-fertilizer. ’Biofertilizer+Glucose’ contains Phylazonit bio-fertilizer and 0,1 g of added glucose. DOI, 10.5256/f1000research.12936.d182664 ^38^.
