# Supplementary material for: Unexpected results in Chernozem soil respiration while measuring the effect of a bio-fertilizer on soil microbial activity
Source: F1000Res. 2017 Dec 18;6:1950. Originally published 2017 Nov 3. [Version 2] doi: 10.12688/f1000research.12936.2 (PMC5747331; doi:10.12688/f1000research.12936.2)
Supplement: Peer review history

# Peer review: Referee response for version 2

**DOI:** 10.5256/f1000research.14500.r29290

**Published:** 2017-12-22

**Authors:** Muhammad Aslam Ali

**Affiliations:** 1 Department of Environmental Science, Bangladesh Agricultural University, Mymensingh, Bangladesh

I have read this submission. I believe that I have an appropriate level of expertise to confirm that it is of an acceptable scientific standard to publish as a research note, however in case of a full manuscript it needs more scientific investigation on the variation of soil respiration as well as CO2 fluxes and soil microbial activities under specified environmental conditions.

I have read this submission. I believe that I have an appropriate level of expertise to confirm that it is of an acceptable scientific standard.

# Peer review: Referee response for version 1

**DOI:** 10.5256/f1000research.14027.r27583

**Published:** 2017-11-29

**Authors:** Muhammad Aslam Ali

**Affiliations:** 1 Department of Environmental Science, Bangladesh Agricultural University, Mymensingh, Bangladesh

1. Why did the authors select Phylazonit biofertilizer?  Does it contain any methanotrophs bacterial spp. or any electron acceptors? Didn’t find the composition.

 2. Why not investigate the CO2 production rate with varying levels such as 0.5%, 1% and 5% substrates application in soils?

 2. What were the initial content of organic carbon, total nitrogen, soil pH, redox status (Soil Eh) and microbial composition of the collected 24 soil samples?

 3. How did the researchers control the pressure within the glass bottles during the experimental period?

 4. How did the authors maintain moisture levels or water filled pore space uniformity in the 24 soil samples containing glass bottles?

 5. Why didn’t you collect the gas samples evolved from the soils in glass bottles at varying time hours?

 6. Why didn’t you follow the light/dark conditions in the Incubator where the glass bottles were kept?

 7. All the Figures are not clear, no contrasting colors or bullets with lines used to differentiate the treatments.

 8. How were soil microbial activities assessed? Methanogens and methanotroph’s relative intensity were not found in this script, which are the major focus related to the current research topic.

I have read this submission. I believe that I have an appropriate level of expertise to confirm that it is of an acceptable scientific standard, however I have significant reservations, as outlined above.

# Response

**Published:** 2017-12-01

**Authors:** Gabriela Bautista

Dear Prof. Muhammad Aslam Ali

 We are trying to answer your questions, and submit a second version of the manuscript in order to clarify the following points.

**1. Why did the authors select Phylazonit biofertilizer?  Does it contain any methanotrophs bacterial spp. or any electron acceptors? Didn’t find the composition.**

The Phylazonit Ltd. provided the biofertilzer for test.

The Methods chapter begins with the information related to the composition: " *Bacillus megaterium*, *Bacillus circulans*, *Pseudomonas putida*, in an optimized ratio for soil injection". We did not say that the fertilizer contains methanotrophs bacterial spp. or any electron acceptors. Thats why the results presented in the paper are unexpected.

**2. Why not investigate the CO2 production rate with varying levels such as 0.5%, 1% and 5% substrates application in soils?**

This Research note discusses only unexpected results come from an experiment that was carried out using Oxitop devices. This is part of a project in which more methods are applied. In an other method (using liquid-alkaline absorption) is possible to setup the different levels, but that is not part of the discussion of the present paper. Using Oxitop bottles only one level is possible for the setup.

**2. What were the initial content of organic carbon, total nitrogen, soil pH, redox status (Soil Eh) and microbial composition of the collected 24 soil samples?**

In the Dataset 1: Average values for a number of different soil properties you can find the main phyical, chemical and microbial soil properties such as pH (H2O), pH (KCl), Organic carbon. We will extend the dataset with the Total Nitrogen in the second version of the paper.

**3. How did the researchers control the pressure within the glass bottles during the experimental period?**

The Oxitop automatically measures the changes in the bottles due to gas consumption by its sensor, there is no needed to apply external measurement.

**4. How did the authors maintain moisture levels or water filled pore space uniformity in the 24 soil samples containing glass bottles?**

The measurement was carried out in closed system (bootles), it is not possible to open the bottles during the measurement.

**5. Why didn’t you collect the gas samples evolved from the soils in glass bottles at varying time hours?**

The Oxitop continously measures the changes. As Fig.1 and Fig.2 show during 168 hours the gas  oxygen consumption/ CO2 production were measured.

**6. Why didn’t you follow the light/dark conditions in the Incubator where the glass bottles were kept?**

In order to avoid the effect of the photosynthesis by algeas. We were interested in soil bacteria activities only.  

**7. All the Figures are not clear, no contrasting colors or bullets with lines used to differentiate the treatments.**

 We do not understand this question. In Both figures we used different colors and bullets. 

- Control (absolute): Blue
- Control + glucose: Red
- Treated: Green
- Treated + glucose: Purple 

**8. How were soil microbial activities assessed? Methanogens and methanotroph’s relative intensity were not found in this script, which are the major focus related to the current research topic.**

This paper was submitted as a Research note. Research notes are often *preliminary studies*, descriptions of unexpected and perhaps unexplained observations or lab protocols. We concluded that "Further genomics research could detect the bacterial strains that consumed the *CO*2 in this soil."

**Gabriela Bautista, Bence Mátyás**

# Peer review: Referee response for version 1

**DOI:** 10.5256/f1000research.14027.r27580

**Published:** 2017-11-20

**Authors:** Ankit Singla

**Affiliations:** 1 Regional Centre of Organic Farming, Bhubaneswar, Odisha, India

Bautista and Matyas observed unexpected results in Chernozem soil respiration following the different fertilizer treatments. I think, the values of Dataset 2 could be directly included in the main content of the paper, if possible. The title of Dataset 1 should be "Average values for various properties of Chernozem soil". 

 I have answered ‘partly’ to the question ‘ *Are all the source data underlying the results available to ensure full reproducibility?’ *as soil ecosystems are very diverse and results could vary under different environmental conditions.

I have read this submission. I believe that I have an appropriate level of expertise to confirm that it is of an acceptable scientific standard.
